# Examining the link between 179 lipid species and 7 diseases using genetic predictors

**DOI:** 10.1016/j.ebiom.2025.105671

**Published:** 2025-03-28

**Authors:** Linda Ottensmann, Rubina Tabassum, Sanni E. Ruotsalainen, Mathias J. Gerl, Christian Klose, Daniel L. McCartney, Elisabeth Widén, FinnGen, Kai Simons, Samuli Ripatti, Veronique Vitart, Caroline Hayward, Matti Pirinen

**Affiliations:** a Institute for Molecular Medicine Finland, HiLIFE, University of Helsinki, Helsinki, Finland; b Centre for Genomic and Experimental Medicine, Institute of Genetics and Cancer, University of Edinburgh, Western General Hospital, Edinburgh, United Kingdom; c Lipotype GmbH, Dresden, Germany; d Department of Public Health, Clinicum, Faculty of Medicine, University of Helsinki, Helsinki, Finland; e Broad Institute of the Massachusetts Institute of Technology and Harvard University, Cambridge, MA, USA; f Medical Research Council Human Genetics Unit, Institute of Genetics and Cancer, University of Edinburgh, Western General Hospital, Edinburgh, United Kingdom; g Department of Mathematics and Statistics, University of Helsinki, Helsinki, Finland

**Keywords:** Lipidomics, GWAS, PGS, Mendelian randomisation, Disease risk

## Abstract

**Background:**

Genome-wide association studies of lipid species have identified several loci shared with various diseases, however, the relationship between lipid species and disease risk remains poorly understood. Here we investigated whether the plasma levels of lipid species are causally linked to disease risk.

**Methods:**

We built genetic predictors of 179 lipid species, measured in 7174 Finnish individuals, by utilising either 11 high-impact genomic loci or genome-wide polygenic scores (PGS). We assessed the impact of the lipid species on seven diseases by performing disease association across FinnGen (n = 500,348), UK Biobank (n = 420,531), and Generation Scotland (n = 20,032). We performed univariable Mendelian randomisation (MR) and multivariable MR (MVMR) analyses to examine whether lipid species impact disease risk independently of standard lipids.

**Findings:**

PGS explained >4% of the variance for 34 lipid species but variants outside the high-impact loci had only a marginal contribution. Variants within the high-impact loci showed association with all seven diseases. MVMR supported a causal role of ApoB in ischaemic heart disease after accounting for lipid species. Phosphatidylethanolamine-increasing *LIPC* variants seemed to lower age-related macular degeneration risk independently of HDL-cholesterol. MVMR suggested a protective effect of four lipid species containing arachidonic acid on cholelithiasis risk independently of Total Cholesterol.

**Interpretation:**

Our study demonstrates how genetic predictors of lipid species can be utilised to gain insights into disease risk. We report potential links between lipid species and age-related macular degeneration and cholelithiasis risk, which can be explored for their utility in disease risk prediction and therapy.

**Funding:**

The funders had no role in the study design, data analyses, interpretation, or writing of this article.

**Research in context**

**Evidence before this study**

Studies assessing the link between lipid species and diseases have been limited to specific metabolite–disease combinations. Hence, the relationship between lipid species and disease risk remains poorly understood.

**Added value of this study**

In this study, we present the findings of a comprehensive investigation of causal link between 179 lipid species and 7 diseases from different disease groups. We confirm the previously reported causal role of ApoB in ischaemic heart disease even after accounting for more detailed lipid species in multivariate analysis. We show that *LIPC* variants increase Phosphatidylethanolamines and lower age-related macular degeneration risk independently of HDL-cholesterol. We identified four lipid species containing arachidonic acid with a potential protective effect on cholelithiasis risk independently of Total Cholesterol.

**Implications of all the available evidence**

These findings provide important leads about the lipids-disease links that could be explored further for their utility in disease risk prediction and treatment. For example, our findings suggest potential links between lipid species and age-related macular degeneration and cholelithiasis risk.

## Introduction

The standard lipids, including high-density lipoprotein cholesterol (HDL-C), low-density lipoprotein cholesterol (LDL-C), triglycerides (TG), and total cholesterol (TC), are routinely measured to assess disease risk.^1^ More advanced lipidomics technologies allow us to measure hundreds of circulating lipid species, such as ceramides (Cer), lysophosphatidylcholines (LPC), phosphatidylcholines (PC), sphingomyelins (SM), and triacylglycerols (TAG), which might outperform the standard lipid measurements in cardiovascular disease risk assessment.^2^, ^3^, ^4^, ^5^, ^6^, ^7^, ^8^, ^9^, ^10^, ^11^, ^12^, ^13^

Genome-wide association studies (GWAS) of lipid species have identified many loci,^14^, ^15^, ^16^, ^17^, ^18^, ^19^, ^20^, ^21^, ^22^, ^23^, ^24^, ^25^, ^26^, ^27^, ^28^, ^29^, ^30^, ^31^, ^32^, ^33^, ^34^, ^35^, ^36^ which are also risk loci for diseases,^20^^,^^33^^,^^36^, ^37^, ^38^ including cardiovascular disease,^20^^,^^33^ age-related macular degeneration (AMD),^37^^,^^38^ and cholelithiasis (gallstones).^20^ However, the relationship between lipid species and disease risk remains poorly understood. Identifying causal links between lipid species and disease could provide a potential for new therapies for the disease via modification of the lipid levels.

Findings from GWAS of lipid species in the general population can be utilised to generate such genetic predictors of lipid levels that are not influenced by disease processes. Thus, the association between genetically predicted lipid levels and diseases can suggest causal links in a way that avoids reverse causation from the disease process to the lipid levels. Another advantage of genetic predictors is that they can be evaluated on large biobank collections that already include both genetic and disease information but where no lipid species quantification is available. To generate genetic predictors, we can either focus on individual variants or on polygenic scores (PGS) that combine all variants into a single predictor.

The variants with a high impact on a lipid species can inform on a possible causal effect on a disease since the statistical power to detect a disease association should be high if causality holds true and if the lipid species is a major contributor to the disease. On the other hand, any single genetic association between a lipid species and a disease may also emerge due to a shared pathway instead of a causal link. The Mendelian randomisation (MR) approach^39^ is a way to jointly utilise multiple variants to assess the plausibility of a causal link between genetically predicted exposures, such as lipid levels, and an outcome, such as a disease. In univariable MR, where each exposure is studied separately, the high phenotypic correlation within the lipidome hinders the causal interpretation. Multivariable MR (MVMR)^40^^,^^41^ has been suggested to solve the issue of correlated exposures when all relevant exposures are included in the analysis. For standard lipids, MVMR studies report an effect of apolipoprotein-B (ApoB) on coronary heart disease independently of LDL-C and TG.^42^^,^^43^ For AMD, a causal effect of HDL-C levels has been suggested by MVMR.^44^ For cholelithiasis (gallstones), one MVMR study reported a significant effect of TC independently of LDL-C^45^; another study for the related trait cholecystitis (inflammation of the gallbladder) reported a significant effect of LDL-C but not TC.^46^

To our knowledge, the only MR studies including lipid species measured by mass spectrometry were performed for the METSIM cohort^47^ where plasma metabolites were measured for 6136 Finnish men. Univariable MR indicated a protective effect of ten phosphatidylethanolamine species on AMD^48^ and an increased risk for gallbladder disorders by campesterol^47^^,^^48^ and two phosphatidylcholine species.^48^

PGS, combining effects of variants across the genome,^49^ can provide stronger genetic predictors than individual variants. This is beneficial for both detecting associations between exposures and diseases and for studying the dose-response relationship between the two. However, an association between a PGS of an exposure and a disease provides less evidence for a direct causal link than the MR approach. Additionally, given the relatively small sample size of the current lipid species GWAS, it is unclear how powerful PGS can be created and how their predictive power compares to that of the individual loci.

**Fig. 1:**
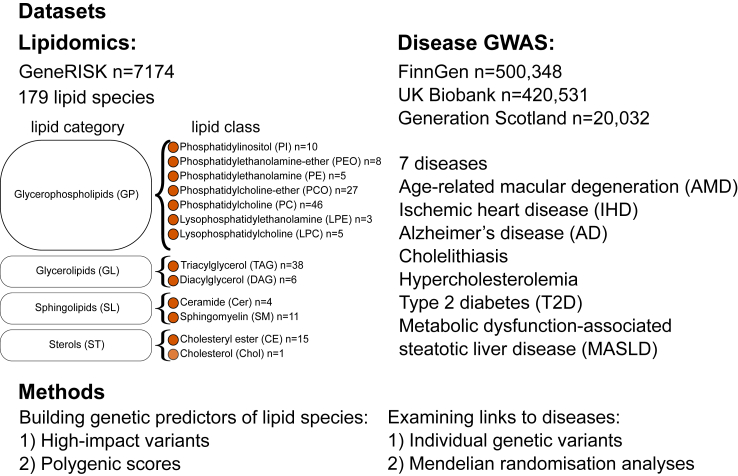
**Study design.** The 179 lipid species belong to 13 lipid classes and 4 categories. Lipid class, category, SwissLipidsName, and GWAS sample size of the lipid species are listed in Supplementary Table S1.

In this work, we study whether the levels of lipid species are causally linked to diseases. For this, we built genetic predictors for 179 lipid species belonging to 13 classes (Supplementary Table S1) covering 4 categories: glycerolipids, glycerophospholipids, sphingolipids, and sterols. Lipid species levels measured by mass spectrometry were available for plasma samples of 7174 Finnish individuals from the GeneRISK cohort and the corresponding GWAS have been previously described.^36^ We considered two ways to build genetic predictors of the lipid species: utilising only variants in loci with a high impact and generating PGS. We evaluated the associations between genetically predicted lipid species levels and seven diseases representing cardiovascular disease (ischaemic heart disease (IHD)), hyperlipidaemia (pure hypercholesterolaemia), diabetes (type 2 diabetes (T2D)), diseases of the digestive system (cholelithiasis, metabolic dysfunction-associated steatotic liver disease (MASLD)), neurological disease (Alzheimer’s disease (AD)), and diseases of the eye (AMD). So far, lipid species measured by mass spectrometry have been utilised in MR studies only for specific metabolite–disease combinations.^47^^,^^48^ Here, we perform more comprehensive univariable and multivariable two-sample MR analyses for 179 lipid species and the 7 diseases to assess the causal link between the lipid species and the diseases (Fig. 1).

## Methods

### Cohorts with lipidome profiles

#### GeneRISK

For the main analyses, data from the GeneRISK cohort^50^ including 7342 participants recruited from Southern Finland during 2015–2017 at the age of 45–66 years was utilised. Further details about the recruitment process are described in Supplementary Note S1. The basic characteristics of the GeneRISK study cohort are listed in Supplementary Table S2. Lipidome profiling was performed for 7302 participants and 7276 individuals remained after quality control. The biological samples (DNA, blood, serum, plasma) and the participants’ demographic information and health data, genetic data, and lipidomic data are stored in the THL Biobank [https://thl.fi/en/web/thl-biobank/for-researchers/sample-collections/generisk-study]. The GeneRISK study was carried out according to the principles of the Helsinki declaration and the Council of Europe’s (COE) Convention of Human Rights and Biomedicine. All study participants gave their informed consent to participate in the study. The study protocols were approved by The Hospital District of Helsinki and Uusimaa Coordinating Ethics committees (approval No. 281/13/03/00/14 (GeneRISK)).

Genotyping was performed using the Illumina HumanCoreExome BeadChip and genotype calling was done with GenomeStudio and zCall at the Institute for Molecular Medicine Finland (FIMM). Genotypes were lifted over to the human genome build version 38 (GRCh38/hg38) according to the protocol described in [https://doi.org/10.17504/protocols.io.nqtddwn]. For estimation of genetic ancestry, principal component analysis (PCA) was performed using 61,106 good quality (missingness <10%, minor-allele frequency (MAF) ≥ 0.05, and Hardy–Weinberg equilibrium *P*-value (HWE) > 1e-6) and approximately independent (LD pruning with PLINK v1.9: *r*^2^ threshold of 0.2, window size 50 kb, step size 5) genetic variants. Individuals with non-Finnish genetic ancestry estimated based on the PCA or birthplace obtained from the questionnaire were removed. Samples born in Estonia, Russia, and Sweden, but clustered with the samples of Finnish ancestry in PCA, were retained. Samples (*N* = 30) with extreme heterozygosity (beyond ± 4 s d) were excluded. After the quality control, 7174 samples, consisting of 4579 females and 2595 males, with both genotype and lipidome data were considered for the subsequent analyses.

Pre-phasing of genotype data was performed with Eagle v2.3.5^51^ with the number of conditioning haplotypes set to 20,000. Imputation of genotypes was done with Beagle v4.1^52^ using the population-specific Sequencing Initiative Suomi (SISu) v3 reference panel based on high-coverage (25–30x) whole-genome sequences for 3775 Finnish individuals. The procedure is described in [https://doi.org/10.17504/protocols.io.nmndc5e]. Variants with imputation INFO score <0.70 and MAF < 0.01 were excluded and 12,776,997 variants remained.

#### FINRISK

For a validation of the lipid species PGS, we utilised a subset of the Finnish National FINRISK study.^53^ The FINRISK study collection is a population-based survey that has been conducted every 5 years since 1972. Collections from the 1992, 1997, 2002, 2007, and 2012 surveys are stored in the THL Biobank. The FINRISK study was conducted according to the principles of the Helsinki declaration. Written informed consent was obtained from all the study participants. The study protocols were approved by the ethics committees of the participating centres (The Hospital District of Helsinki and Uusimaa Coordinating Ethics committees, approval No. 184/13/03/00/12).

Genotyping was performed using the Illumina HumanCoreExome BeadChip. The genotype calls were generated using zCall at the Institute for Molecular Medicine Finland (FIMM). Samples with low call rate (<95%), sex discrepancies, excess heterozygosity, and non-European ancestry were excluded. Variants with low call rate (<95%) and deviation from Hardy–Weinberg Equilibrium (HWE *P* < 1e-6) were removed. Imputation was performed using IMPUTE2.^54^ Variants with imputation INFO score <0.70 were excluded. For subsequent analyses, ∼9.3 million variants with MAF >0.005 were available.

For 1142 samples randomly selected from the FINRISK 2012 survey lipidomic profiling was available. Samples with low lipid content were removed and 1080 samples remained. For 1032 samples (476 male sex and 556 female sex), genotype data was available. These samples were utilised to validate the PGS. The samples were included in a GWAS published previously^20^ but they were not included in the GWAS^35^ that we used for generating the PGS.

### Lipidomic profiling

Mass spectrometry-based lipid analysis for GeneRISK and FINRISK was performed by shotgun lipidomic analysis at Lipotype GmbH (Dresden, Germany). Direct infusion in a QExactive mass spectrometer from Thermo Scientific with a TriVersa NanoMate ion source from Advion Biosciences was utilised for sample analysis.^55^ The lipidomics data were analysed using lipid identification software and a data management system developed by Lipotype GmbH.^56^^,^^57^

### Genome-wide association studies of lipids in GeneRISK

GWAS of lipid species, published by us previously,^36^ were performed using the linear-mixed-model software MMM v1.01 on inverse-normal transformed residuals adjusted for age, sex, collection site (clinic), lipid medication, first ten principal components of genetic population structure, and ancestry (separate indicator variables for individuals born in Russia, Estonia, and Sweden).

Samples with missing values for a specific lipid species were excluded in the GWAS for that lipid species and therefore the number of samples per GWAS ranged between 5287 and 7174. We report two-sided *P*-values.

GWAS of standard lipids in GeneRISK were performed with the same method and same covariate adjustments as the GWAS of the lipid species described above.

### Calculation of polygenic scores

We derived PGS for 131 lipid species using GWAS summary statistics from the GeneRISK cohort for 7174 individuals by two approaches: (1) PRS-CS,^58^ which employs Bayesian regression and continuous shrinkage priors, and (2) by clumping and *P*-value thresholding as implemented in PLINKv1.9. Both PGS methods resulted in variant-specific weights by which PGS can be computed for additional samples. The PGS were computed for only those 131 lipid species that were measured in the FINRISK cohort (n = 1032),^20^ which is independent of the GeneRISK cohort and was used for validation.

We split the FINRISK cohort into a validation set (n = 772) and a test set (n = 257) and removed related individuals. To select the shrinkage parameter phi for PRS-CS we performed analysis for 19 traits with 8 different values of phi (1, 1e-2, 1e-4, 1e-5, 1e-6, 1e-7, 1e-8) and selected the value which led to the largest R^2^ value (1e-7) within the validation set of the FINRISK cohort. PGS calculation with PLINKv1.9 was performed with parameters --clump-p1 1 --clump-r2 0.1 --clump-kb 250. *P*-value thresholding was performed with the lower bound 0 and the following *P*-value upper bounds (5e-8, 1e-6, 1e-4, 0.01, 0.05, 0.1, 0.5). The *P*-value upper bound with the best fit in the validation set was chosen. All results about variance explained by PGS are reported for the test set of the FINRISK cohort.

### Identification of high-impact loci

Regional heritability for 179 lipid species from the GeneRISK cohort was estimated with FINEMAP v1.4.^59^^,^^60^ The fine-mapping was performed for each genome region that showed a genome-wide significant association in our previous GWAS of these traits.^36^ We computed the in-sample linkage disequilibrium (LD) matrix using LDstore2^61^ from the genotype dosages. The maximum number of causal variants per region was set to ten. We determined the number of independent association signals for each region by the number of informative credible sets (CS) among those CS for which FINEMAP gave the highest posterior probability. We considered a CS informative if the minimum *r*^2^ among its variants was ≥0.1. The top variant from each CS was chosen to represent the association signal except if the CS contained functional variants in high LD (*r*^2^ > 0.95) with the top variant, in which case the functional variant with the largest *r*^*2*^ to the top variant was chosen.^62^ The MHC region (chr 6: 25–34 Mb) was excluded from the fine-mapping.

Regional heritability for the standard lipids HDL-C, LDL-C, TC, and TG were calculated by fine-mapping UK biobank (UKBB) European ancestry GWAS summary statistics from the Pan-UKBB Project^63^(preprint) using in-sample LD of 343,640 unrelated White British participants (UKBB application number 22627) and the same fine-mapping parameters as for the lipid species.

We defined high-impact loci as the loci reaching a regional heritability value > 2% for at least one lipid species. The locus boundaries and locus names were defined as in our previous study.^36^ Briefly, the overlapping association regions were combined across the traits into a single physical locus. The loci were named by the closest gene to the variant with the lowest *P*-value across the associated traits. If there was a missense variant among the top variants, the locus was exceptionally named by the gene corresponding to the missense variant (for instance for the *FADS2* locus).

We report the genes located in the region 300 kb up- or downstream of the lead variant using Variant Effect Predictor v103.1^64^ and sort the genes according to the distance from the variant to the transcription start site. We assign candidate causal genes to the loci based on candidate gene assignments of previous metabolite GWAS.^34^^,^^35^^,^^47^

### Cohorts with disease information

In this study, we utilised three independent cohorts with disease information for disease association analysis of lipid species variants: FinnGen^65^ (n = 500,348), UK Biobank European ancestry subset (n = 420,531) from the Pan-UKBB project^63^(preprint), and Generation Scotland: Scottish Family Health Study (GS:SFHS)^66^ (n = 20,032).

The disease GWAS utilised in this study are of individuals of European ancestry as determined by genetically estimated ancestry. The respective cohorts’ ancestry determination process, genotyping, imputation, quality control, and GWAS method are listed in Supplementary Note S1.

### Disease associations

We studied disease associations of the 141 lead variants of the lipid species GWAS or representative variants from fine-mapping analysis^36^ in the 3 independent cohorts: FinnGen, UK Biobank, and Generation Scotland.

We considered the following seven diseases: AD, AMD, cholelithiasis, IHD, MASLD, pure hypercholesterolaemia, and T2D. These disease endpoints, except AMD were selected based on a previous phenome-wide analysis across 953 disease endpoints of lipid species-associated variants in FinnGen^36^ as representatives of cardiovascular disease, hyperlipidaemia, diabetes, diseases of the digestive system, and neurological disease. We chose the endpoints to represent the disease groups by balancing the effective sample size and the clarity of the endpoint definition. We included AMD to represent the diseases of the eye because many lipid loci have been reported to be associated with AMD.^38^

To confirm the validity of our data, we considered statin medication as an additional endpoint that we expect to be a proxy for hypercholesterolaemia. While there are other possible reasons for prescribing statins, we assume that most individuals are prescribed statins due to hypercholesterolaemia. The number of cases of the statin medication in FinnGen is significantly larger (n = 183,578) compared to pure hypercholesterolaemia (n = 42,985). The genetic correlation of the two endpoints in FinnGen is 1.00 with 95% CI: [0.95–1.05].

For AD, AMD, cholelithiasis, IHD, MASLD, pure hypercholesterolaemia, and T2D, the FinnGen endpoints with codes G6_ALZHEIMER, H7_AMD, K11_CHOLELITH, I9_IHD, NAFLD, E4_HYPERCHOL, and T2D were used, respectively. MASLD has been recently recommended by a consensus statement^67^ to replace the term non-alcoholic fatty liver disease (NAFLD). While the diagnosis criteria of MASLD and NAFLD differ, there exists a large sample overlap between the two diagnoses. We have used the ICD-10 code K76.0 (NAFLD) to define MASLD cases as this is currently the most suitable ICD-10 code.^68^ For the statin medication, the FinnGen endpoint with code RX_STATIN was used. FinnGen endpoints were constructed from register codes from the following national health registers: hospital and outpatient visits (HILMO), primary health care (AvoHILMO), cause of death, reimbursed medication entitlements, prescribed medicine purchases, and the Finnish Cancer Registry.^65^ The definitions of FinnGen endpoints are available at https://www.finngen.fi/en/researchers/clinical-endpoints.

The UKBB Phecodes/ICD-10 codes from Pan-UKBB analysis matching best with the FinnGen endpoints according to the overlap in ICD-10 codes were obtained by utilising similarity scores from https://github.com/FINNGEN/pan-ukbb-mapping. The similarity scores are defined as the size of the unique FinnGen ICD10 codes’ and unique Phecode ICD10 codes’ intersection divided by the size of their union. These scores were used for a previously published meta-analysis of FinnGen and the UKBB.^65^ The similarity scores were 1 for hypercholesterolaemia (272.11), AD (G30), cholelithiasis (574.1) and T2D (E11), 0.84 for IHD (411), and 0.25 for MASLD (571.5), with UKBB ICD or Phecode listed in parentheses. For AMD, the Phecode 362.2 defined as Degeneration of macula and posterior pole of retina was used. For statin medication, Phecode HMG_CoA_reductase_inhibitor|statin was used.

Diseases in Generation Scotland were defined based on Phecodes or ICD-10 codes from Pan-UKBB analysis matching best with the FinnGen endpoints. For T2D, individuals with diabetes medication (BNF section Drugs used in Diabetes) were excluded from controls. For hypercholesterolaemia and AMD, the case definition according to the UKBB Phecodes used for case definition in UKBB GWAS led to <50 cases, and these diseases were defined according to the most specific ICD-10 codes used for the case definition in FinnGen, for which >50 cases were identified (E78 Disorders of lipoprotein metabolism and other lipidaemias and H35 Other retinal disorders, respectively). For statin medication, prescription data for ATC code C10AA was utilised. Of note, the prescription data was limited to the participants in the Tayside region until ∼2009. Additionally, self-reported information at baseline was used to identify participants on statin medication.

The number of cases and controls for the diseases and the phecodes/ICD10 codes utilised for case and control definitions are given in Supplementary Table S3. Variant-disease associations for 11 high-impact loci reaching the Bonferroni-corrected *P*-value threshold of *P* < (0.05/7 = 7.14e-3), correcting for the number of diseases, are listed in Supplementary Data S1. Variant-disease associations for these loci with statin medication are given in Supplementary Data S1.

### Univariable mendelian randomisation

Two-sample Mendelian Randomisation (MR) was run for pairs of lipid species or standard lipids from GeneRISK (exposure) and disease endpoints from FinnGen (outcome). Both cohorts consist of individuals of Finnish genetic ancestry. The FinnGen cohort (n = 500,348) consists of legacy cohorts including GeneRISK (n = 7174) and prospective samples. Therefore, a small proportion of FinnGen disease endpoint cases or controls may overlap with GeneRISK samples.

We performed MR with the following methods: Inverse Variance Weighted (IVW) using a random-effects model, Weighted Median, MR-Egger, and MR Lasso from the MendelianRandomization R-package v0.9.0 and MR-PRESSO^69^ from the MR-PRESSO R-package v1.0. Genetic variants were selected as instrumental variables (IV) by LD clumping the GWAS SNPs with MAF >0.01 using PLINK v1.9 with in-sample LD and parameters r^2^ 0.01, distance 10.000 kb, and *P*-value threshold 5e-8. Palindromic SNPs with MAF >0.42 were removed.

MR was performed for the lipid species or standard lipids with >4 genetic instruments (57 traits). Due to the high phenotypic correlation between the 57 lipids, we performed principal component analysis of the mean imputed phenotypes and found that 22 principal components explain >90% of the total variance of the 57 traits. The phenotypic correlations are listed in Supplementary Data S2 and visualised as a heatmap in Supplementary Fig. S1. Results were considered significant if the Bonferroni-corrected threshold of 2.3e-3 (0.05/22) was reached in IVW-MR and had *P* < 0.05 and the consistent effect direction for all MR methods. Additionally, we report which results reach the more stringent threshold 3.25e-4 (0.05/22∗7) in IVW-MR, where the significance level has been corrected for the number of outcomes (7 diseases). All MR results and test statistics are listed in Supplementary Data S2.

A genetic variant is a valid IV if it meets the following assumptions: it must be (IV1) associated with the exposure (the ‘relevance’ assumption); (IV2) independent of all confounders of the exposure and the outcome (the ‘exchangeability’ assumption); and (IV3) independent of the outcome given the exposure (the ‘exclusion restriction’).^41^^,^^70^ We performed several sensitivity analyses to assess the validity of the MR assumptions. Genetic variants should strongly predict the exposure to satisfy assumption IV1. We calculated *F*-statistics and ensured that all genetic variants selected as IVs by LD clumping have an *F*-statistic >10 to avoid weak instrument bias.^71^ We report an approximation of the first-stage *F*-statistic for all variants calculated by the IVW MR method based on the summarised data. If the IVs are confounded by variables that also influence the outcome, the assumption IV2 is violated. The presence of horizontal pleiotropy, where the IVs have an effect on the outcome that is not through the exposure, violates assumption IV3.^41^ To assess the pleiotropy of the IVs we obtained heterogeneity test statistics (Cochran’s Q statistic and associated *P*-value) from MR-Egger and IVW MR. The null hypothesis of the heterogeneity test is that all genetic variants estimate the same causal parameter and rejection of the null indicates that at least one variant may be pleiotropic. We additionally assessed pleiotropy with the MR-Egger intercept test and the MR-PRESSO global test.

We performed several MR methods to assess the robustness of MR results to violations of IV assumptions: The Weighted Median MR method gives a consistent estimate of the causal effect when 50% of the weight comes from valid IVs, MR Lasso performs IVW MR only on valid IVs, and MR-PRESSO identifies horizontal pleiotropic outliers and calculates outlier corrected MR estimates. We calculated Wald ratios (ratio between the allelic effect estimate from the GWAS of the outcome and the GWAS of the exposure) for each genetic instrument to compare the causal effect estimated using each variant alone to the MR causal effect estimated using all instruments. Forest plots of single-variant Wald ratios and scatterplots of SNP effects and MR results from all methods for all significant MR results were created with ggplot2 R-package with slightly modified code used in the TwoSampleMR R-package^72^^,^^73^ (Supplementary Fig. S2).

We performed a literature review of previous MR studies of lipids and the selected disease endpoints and summarised the MR results of these previous studies in Supplementary Data S3. We report the MR results according to the STROBE-MR guidelines^74^ and provide the STROBE-MR checklist (Supplementary Note S2).

### Multivariable mendelian randomisation

Multivariable MR (MVMR) has been developed to estimate the independent direct effects of multiple exposures, which are potentially related.^40^ For each exposure included in the model, MVMR estimates the effect of the exposure on the outcome conditional on the other included exposures, which is called the direct effect of the exposure. In contrast, univariable MR estimates the total effect of the exposure including effects that act through any other exposure. The difference between MR estimates and MVMR estimates depends on the relationship between the exposures.^75^ For a genetic variant to be considered a valid IV for MVMR, it needs to fulfil the following assumptions: the variant is associated with at least one of the exposures, the variant is not associated with a confounder of the exposure-outcome associations, and the variant is conditionally independent of the outcome given the exposures and confounders.^40^

MVMR was performed using the method MVMR-IVW from the MVMR R-package.^76^ To assess the strength of IVs in the MVMR analyses we calculated the conditional *F*-statistics with the function strength_mvmr() from the MVMR R-package. To avoid weak instrument bias valid IVs must strongly predict each exposure and jointly predict the exposures.^41^ The conditional *F*-statistics of the significant MVMR results reported in our study were >10 ensuring the strength of the IVs. We additionally performed the weak instrument robust MVMR-Q(het) from the MVMR R-package.

We report heterogeneity test statistics (Cochran’s Q and associated *P*-value) from the MVMR-IVW analysis. We also report a modified form of the Cochran’s Q statistic and associated *P*-value calculated with the pleiotropy_mvmr() function from the MVMR R package. We utilised MVMR-EGGER to correct for the measured and unmeasured pleiotropy^77^ and report heterogeneity test statistics and the intercept estimate from MVMR-Egger. Additionally, we applied the following pleiotropy robust methods: MVMR-Robust (performs well for low levels of pleiotropy), MVMR-Lasso (preferred for moderate to high levels of pleiotropy), and MVMR-Median (valid inference up to moderate levels of pleiotropy).^78^

MVMR was performed for the following disease endpoints with significant univariable MR results for a lipid species and without an obvious direct link to lipid levels: IHD, AMD, and cholelithiasis. For these diseases, we performed MVMR analyses for each lipid species as exposure and included a standard lipid previously suggested to be an independent risk factor for the disease as the second exposure. As instruments, the SNPs utilised in the univariable MR of the lipid species or standard lipid were combined and SNPs remaining after LD clumping using PLINK v1.9 with in-sample LD and parameters *r*^2^ 0.01, distance 10.000 kb were included. All MVMR results and test statistics are listed in Supplementary Data S2.

### Role of funders

The funders had no role in the study design, data analyses, interpretation, or writing of this article.

## Results

### Genetic predictors of lipid species

#### High-impact loci

**Fig. 2:**
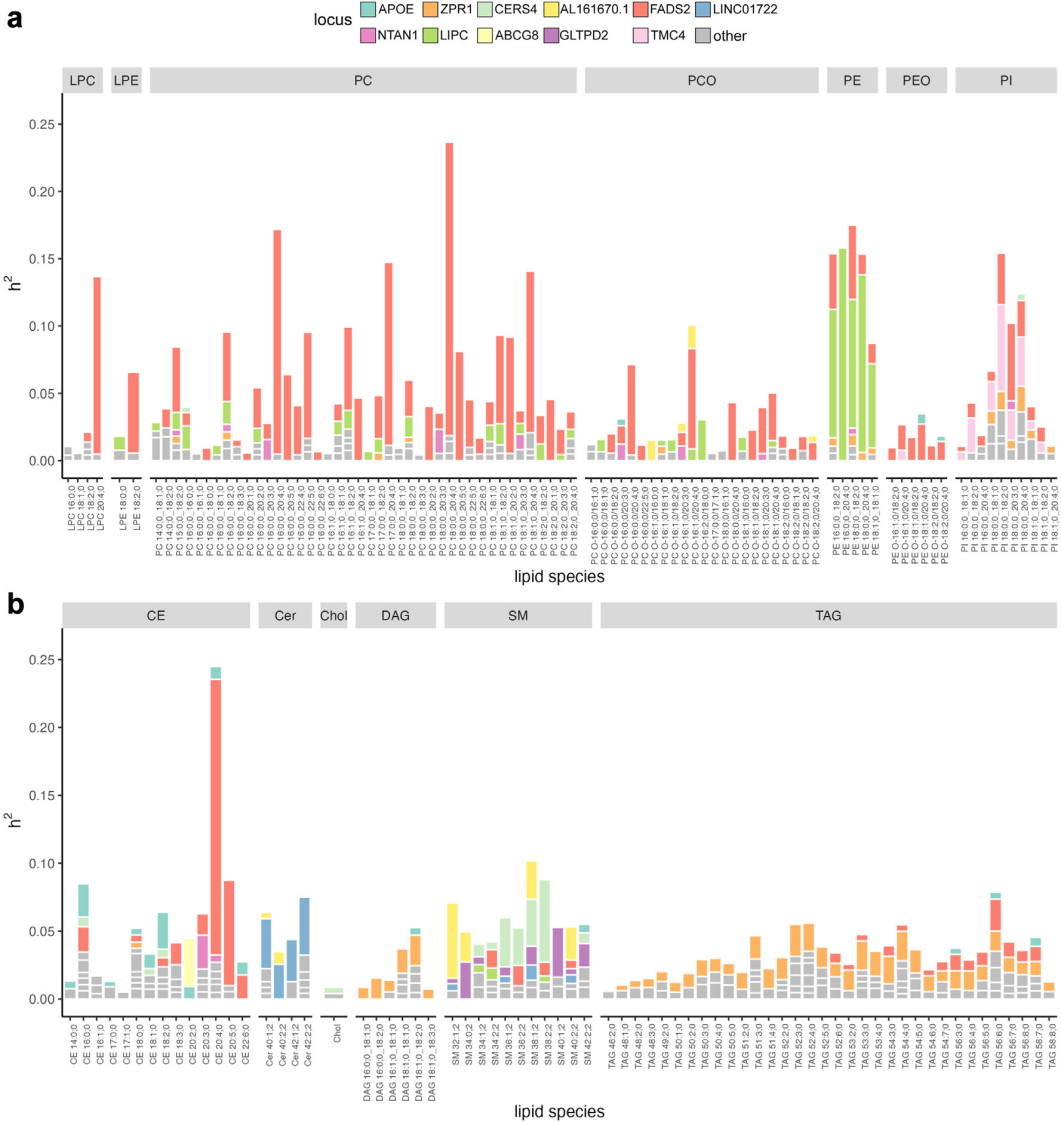
**Regional heritability (*h*^2^) of lipid species for genome-wide significant loci estimated by FINEMAP.** a: glycerophospholipids, b: glycerolipids, sphingolipids, and sterols. High-impact loci (*h*^2^ > 2% for at least one lipid species) are coloured, and the other genome-wide significant loci not among the high-impact loci are depicted as grey. Heritability estimation was performed with FINEMAP using summary statistics of GWAS from n = 7174 biologically independent samples. CE cholesteryl ester, Cer ceramide, DAG diacylglycerol, LPC lysophosphatidylcholine, LPE, lysophosphatidylethanolamine, PC phosphatidylcholine, PCO, phosphatidylcholine-ether, PE phosphatidylethanolamine, PEO phosphatidylethanolamine-ether, PI phosphatidylinositol, SM sphingomyelin, TAG triacylglycerol.

To identify genetic loci with a high impact on the circulating concentrations of lipid species, we obtained regional heritability estimates from fine-mapping analysis of genetic loci associated with lipid species with *P* < 5e-8. These regional heritability estimates extended our previous work where we performed fine-mapping of all genome-wide significant regions^36^ to identify the most probable causal variants. Eleven loci (*AL161670.1*, *CERS4*, *FADS2*, *LINC01722*, *LIPC*, *ZPR1, ABCG8*, *APOE*, *GLTPD2*, *NTAN1*, *TMC4*) reach regional heritability values (*h*^2^) > 2% (Fig. 2). For comparison, we estimated regional heritability for the standard lipids HDL-C, LDL-C, TC, and TG by fine-mapping UKBB White British subset GWAS (Supplementary Fig. S3). We assign potential causal genes to these high-impact loci based on candidate genes from literature considering biological knowledge and we list lipid classes and standard lipids reaching *h*^2^ > 2% and 0.2%, respectively (Table 1, Supplementary Tables S4–S6).

**Table 1:** Genetic loci with a high impact (*h*^2^ > 2%) on the circulating concentrations of lipid species.

Locus	Candidate causal gene(s)	Explanation	Standard lipids with *h*^2^ > 0.2%	Lipid classes of species with *h*^2^ > 2% max. *h*^2^ (trait)
*ABCG8*	*ABCG5*|*ABCG8*	*ABCG5* and *ABCG8* together encode a heterodimeric ATP-dependent transmembrane sterol transporter.	LDL-C, TC	CE 4.5% (CE 20:2; 0)
*AL161670.1*	*SGPP1*	SGPP1 catalyses the degradation of Sphingosine-1-phosphate (S1P) via salvage and recycling of sphingosine into long-chain ceramides. S1P is a bioactive sphingolipid metabolite that regulates diverse biologic processes.		SM 5.6% (SM 32:1; 2)
*APOE*	*APOC1*|*APOC2*|*APOC4*|*APOE*	*APOE/APOC1/APOC2/APOC4* encode a set of related apolipoproteins involved in lipoprotein particle trafficking.	HDL-C, TG	CE 2.7% (CE 18:2; 0)
*CERS4*	*CERS4*	*CERS4* encodes a ceramide synthase which produces ceramides from sphingosine and a long chain acyl CoA, derived from long chain free fatty acids.	LDL-C	SM 6.1% (SM 38:2; 2)
*FADS2*	*FADS1*|*FADS2*	*FADS1* and *FADS2* encode a pair of fatty acid desaturases	TG	CE, LPC, LPE, PC, PCO, PE, PEO, PI, TAG 21.8% (PC 18:0; 0_20:4; 0)
*GLTPD2*	*GLTPD2*	*GLTPD2* encodes a protein homologous to a ceramide-1-phosphate transfer protein, which acts on ceramide-1-phosphate. Ceramide-1-phospate is involved in Sphingolipid metabolism.		SM 3.7% (SM 40:1; 2)
*LINC01722*	*SPTLC3*	*SPTLC3* encodes a subunit of serine palmitoyltransferase, which catalyses the first step in de novo sphingolipid biosynthesis.		Cer 4.3% (Cer 42:2; 2)
*LIPC*	*LIPC*	*LIPC* encodes hepatic triglyceride lipase which hydrolyses triglycerides. The enzyme plays a key role in regulating levels of HDL.	HDL-C, TC	PCO, PE 15.8% (PE 16:0; 0_20:4; 0)
*NTAN1*	*PDXDC1*	Pyridoxal-dependent decarboxylase domain–containing protein 1. Previous study indicates role in the metabolism of 20:2 and 20:3 fatty acids.		CE 2.5% (CE 20:3; 0)
*TMC4*	*MBOAT7*	*MBOAT7* encodes a lysophosphatidylinositol acyltransferase.		PI 6.5% (PI 18:0; 0_18:2; 0)
*ZPR1*	*APOA1|APOA4|APOA5|APOC3*	The *APOA5/APOA4/APOA1/APOC3* gene cluster encodes a set of related apolipoproteins involved in trafficking of apolipoprotein particles.	HDL-C	DAG, TAG 2.4% (TAG 52:3; 0)

CE, cholesteryl ester; Cer, ceramide; DAG, diacylglycerol; LPC, lysophosphatidylcholine; LPE, lysophosphatidylethanolamine; PC, phosphatidylcholine; PCO, phosphatidylcholine-ether; PE, phosphatidylethanolamine; PEO, phosphatidylethanolamine-ether; PI, phosphatidylinositol; SM, sphingomyelin; TAG, triacylglycerol.

Among the 11 high-impact loci, the *FADS2* locus had the highest effect on lipids with *h*^2^ > 2% for 38 lipid species of 9 lipid classes (CE, LPC, LPE, PC, PCO, PE, PEO, PI, TAG) and *h*^2^ > 0.2% for the standard lipid TG. The potential causal genes in the locus, *FADS1* and *FADS2*, encode delta-6 and delta-5 fatty acid desaturases, respectively. For the *ZPR1* and *LIPC* loci, lipid species from two classes, DAG, TAG, and PCO, PE, respectively, reached *h*^2^ > 2%. At the *LIPC* locus, which encodes hepatic triglyceride lipase, we observe especially large regional heritability (6–16%) for lipid species from the class PE, and the standard lipids HDL-C and TC reached *h*^2^ > 0.2%. The *LIPC* locus had the largest number of independent signals (3–8 signals).

For the remaining loci, only species from one lipid class reached *h*^2^ > 2%. Locus *LINC01722* harbouring *SPTLC3*, which encodes a subunit of serine palmitoyltransferase catalysing the first step in de novo sphingolipid biosynthesis, had a high impact only on ceramides. The *GLTPD2*, *CERS4*, and *AL161670.1* loci had a high impact only on sphingomyelins. The nearest genes to the lead variant rs7157785 at *AL161670.1* are *SYNE2* and *AL161670.1* but previous studies indicate *SGPP1*, located 40 kb from rs7157785, as a candidate gene.^22^^,^^28^^,^^32^^,^^35^ *SGPP1* catalyses the degradation of Sphingosine-1-phosphate into long-chain ceramides. The *NTAN1, APOE*, and *ABCG8* loci had a high impact only on cholesteryl esters. At the *ABCG8* locus, only CE 20:2; 0 reached *h*^2^ > 2%, and the standard lipids LDL-C, and TC reached *h*^2^ > 0.2%. *ABCG5* and *ABCG8* are candidate genes at this locus and together they encode a heterodimeric ATP-dependent transmembrane sterol transporter.

**Fig. 3:**
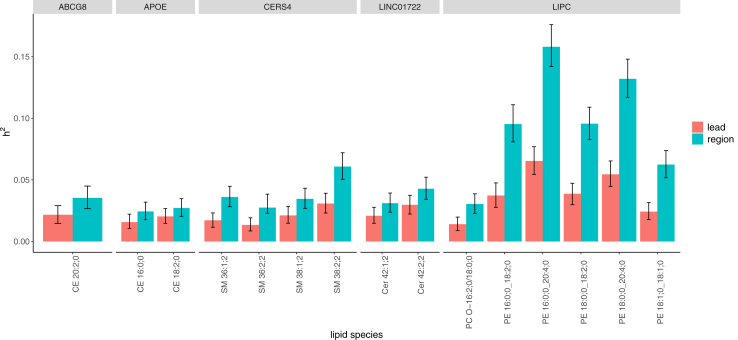
**Comparison of heritability (*h*^2^) estimates of lead variant and regional *h*^2^ estimates for high-impact loci named at the top.** Error bars represent 95% CI. Only the trait-locus combinations for which regional *h*^2^ is located outside the 95% CI of lead variant *h*^2^ are shown. Heritability estimation was performed with FINEMAP using summary statistics of GWAS from n = 7174 biologically independent samples.

We compared the regional heritability estimates of each locus to the heritability estimates of the GWAS lead variant in the locus alone (Fig. 3, Supplementary Data S1). Of the 73 trait-locus pairs with *h*^2^ > 2%, for 15 pairs the regional *h*^2^ estimate exceeded the 95% credible interval (CI) of the heritability of the lead variant. The number of independent signals for these trait-locus pairs was between two and eight.

#### Polygenic scores of lipid species as genetic predictors

To examine the gain of variance explained when adding variants outside the high-impact loci, we created PGS for lipid species. PGS were generated for 131 lipid species (available in both cohorts- GeneRISK and FINRISK) using the GeneRISK^50^ cohort data from 7174 individuals and validated using the FINRISK^53^ cohort (n = 1032). We performed the standard clumping and thresholding PGS calculation with PLINKv1.9, which clumps variants and utilises *P*-value ranges for thresholding. We calculated PGS for the following *P*-value upper bounds (5e-8, 1e-6, 1e-4, 0.01, 0.05, 0.1, 0.5) and selected the best-performing model. We additionally utilised the Bayesian polygenic prediction model PRS-CS,^58^ which incorporates variants across the genome by placing a continuous shrinkage prior on the effect sizes. We observed larger variance explained (R^2^) values in the validation data when the weights generated by PRS-CS were used compared to the weights generated by PLINK for 90 of the 131 lipid species (Supplementary Data S1).

**Fig. 4:**
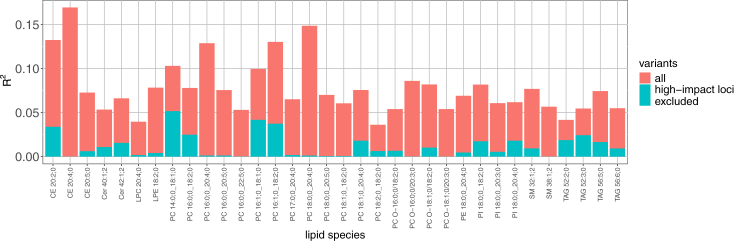
**Variance explained (R^2^) by PGS for 25 lipid species reaching R^2^ > 4% with PRS-CS in the FINRISK validation cohort.** R^2^ for both the full PGS calculation and for the calculation without high-impact loci is given. PGS weights were calculated using summary statistics of GeneRISK GWAS from n = 7174 biologically independent samples. Scores were validated using n = 1032 biologically independent samples from the FINRISK cohort.

PGS, calculated with PRS-CS, explained >4% of the variance for 34 lipid species in the validation cohort with the largest R^2^ (16.9%) reached for CE 20:4; 0. After excluding the regions of the 11 high-impact loci from the calculation, the R^2^ values for the 34 species ranged from 9.3e-5 to 0.052 (Fig. 4).

#### Disease associations

**Fig. 5:**
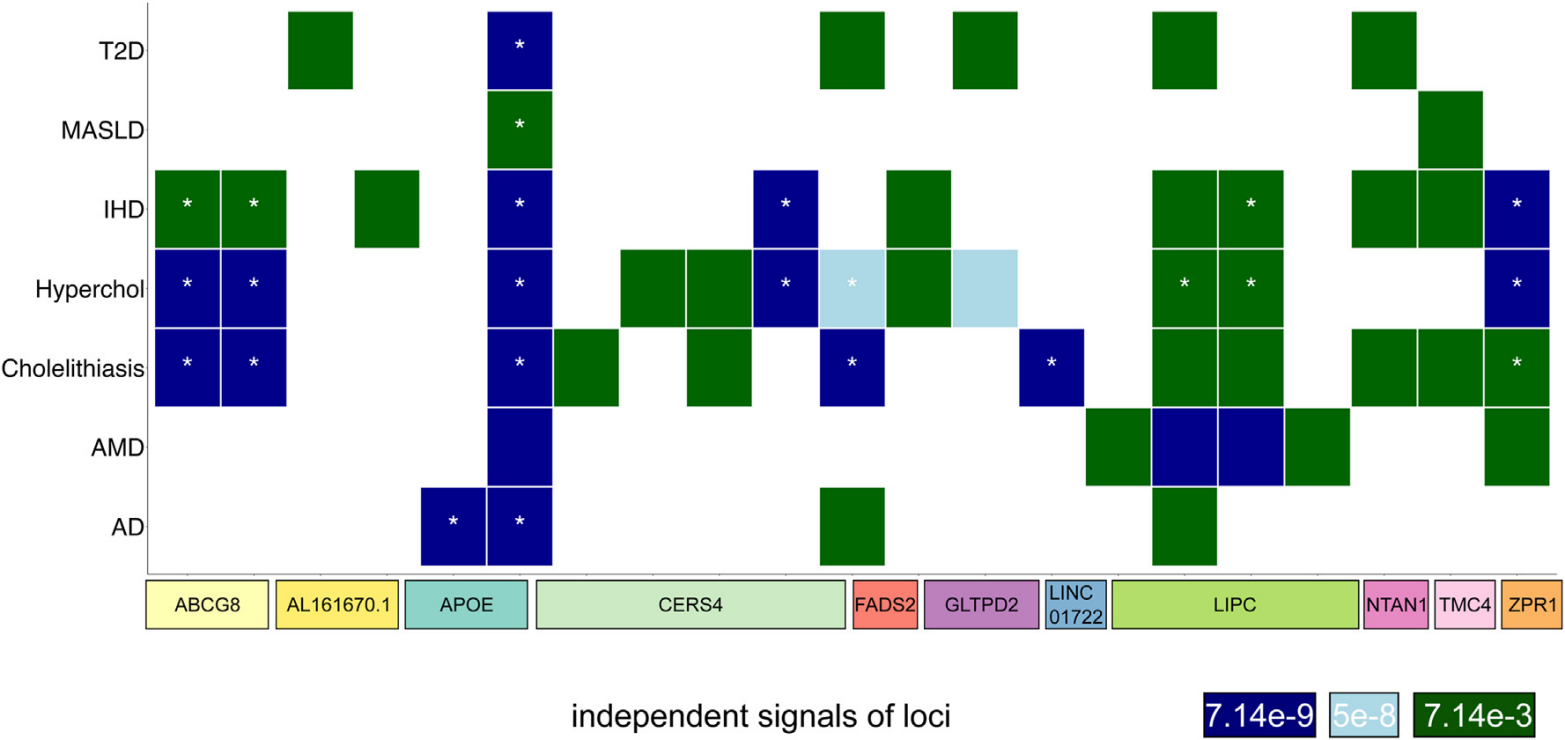
**Disease associations for high-impact loci.** Columns indicate independent signals at the 11 high-impact loci. Rows represent the seven diseases. Coloured tiles depict that at least one lead variant or FINEMAP representative variant from a genome-wide significant lipid species association reaches a specific *P*-value threshold in a disease endpoint GWAS. Two-sided *P*-values were calculated using a linear-mixed-model. Asterisks denote that the threshold 0.05/7 = 7.14e-3 (corrected for the number of diseases) is reached in multiple cohorts. AD: Alzheimer’s disease, AMD: age-related macular degeneration, Hyperchol: hypercholesterolaemia, IHD: ischaemic heart disease, MASLD: Metabolic dysfunction-associated steatotic liver disease, T2D: type 2 diabetes.

To explore the disease relevance of the 11 high-impact lipid-associated loci, we assessed associations of lead variants or representative variants of credible sets from fine-mapping analysis located within these 11 high-impact loci with 7 diseases. We identified 53 variant–disease associations for variants from 21 independent association signals of the 11 high-impact loci at a *P*-value threshold of *P* < 7.14e-3, corresponding to 0.05 corrected for the number of diseases (7) (Fig. 5, Supplementary Data S1). We compared the pure hypercholesterolaemia associations to the association with statin medication, whose case count is over four-fold in FinnGen compared to pure hypercholesterolaemia. As expected, all 46 variants reaching *P* < 5e-8 for pure hypercholesterolaemia reach the same threshold for statin medication (Supplementary Data S1).

### Univariable mendelian randomisation

Univariable MR was performed for each combination of one disease as the outcome and one lipid species or standard lipid measure as the exposure with the MR methods Inverse Variance Weighted MR (IVW-MR), Weighted Median, MR-Egger, MR-Lasso as implemented in MendelianRandomization v.0.9.0, and MR-PRESSO as implemented in MR-PRESSO v.1.0 (Supplementary Data S2). We also performed these MR analyses with statin medication as the outcome. We selected genetic instruments for the exposure by LD clumping GWAS variants with minor allele frequency >0.01 with parameters r^2^ 0.01, distance 10.000 kb, and *P*-value threshold 5e-8. We restricted the analysis to the 57 lipid traits with >4 genetic instruments. We considered the results significant if they reached the multiple testing corrected threshold of 2.3e-3 (0.05/22) in IVW-MR, correcting for the 22 principal components explaining 90% of the total variance of the 57 traits, and had *P* < 0.05 and the consistent effect direction for all MR methods.

We also report which results reach a more stringent threshold 3.25e-4 (0.05/22∗7) in IVW-MR, correcting for the number of outcomes (seven diseases) (Supplementary Data S2).

For MASLD and AD, no results were significant. For T2D, we found only two significant associations: PE 16:0; 0_20:4; 0 and SM 36:1; 2 with IVW-MR *P*-values of 2e-3 and 1e-4, respectively. HDL-C had an MR-IVW *P*-value of 1e-5 but *P* > 0.05 for MR-Egger.

For pure hypercholesterolaemia, results were significant for eight lipid species and four standard lipids. These lipids, except TAG 51:3; 0, were also significant in the MR analysis performed for statin medication. Since for hypercholesterolaemia a direct relationship with cholesterol levels is self-evident we focus here on the MR results of the remaining three diseases – IHD, AMD, and cholelithiasis.

**Fig. 6:**
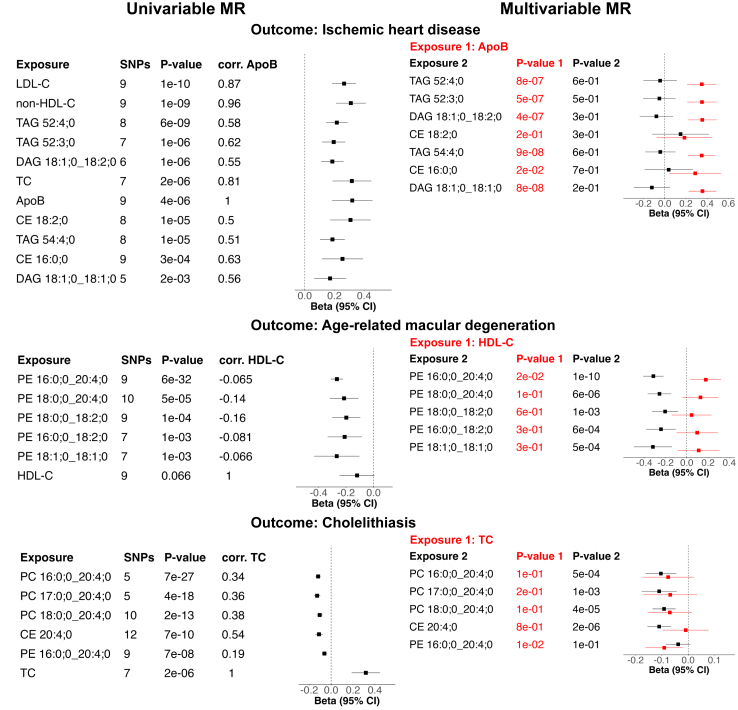
**Forest plots of effect sizes of IVW-MR for IHD, AMD, and cholelithiasis.** Univariable MR results are shown when *P* < 2.3e-3 (0.05/22) in IVW-MR, *P* < 0.05 for all MR methods, and the effect direction is consistent for all methods. Univariable MR results of the standard lipid previously suggested to be causal for the disease are shown (ApoB for IHD, HDL-C for AMD, and TC for cholelithiasis). The pairwise Pearson correlation of the exposure with this standard lipid is listed. Each multivariable MR analysis contains as exposures the standard lipid (Exposure 1, in red) and a lipid species (Exposure 2, in black). Multivariable MR results are shown for the lipid species reaching significance in univariable MR.

For IHD, AMD, and cholelithiasis, seven, five, and five lipid species reached significance, respectively (Fig. 6). Of these, for IHD and AMD, one and two species, respectively, do not reach the more stringent *P*-value threshold of 3.25e-4. We show scatterplots of significant MR results and forest plots comparing the causal effect of each SNP on their own to two methods using all genetic instruments in Supplementary Fig. S2. For IHD, five highly correlated TAG/DAG species: TAG 52:4; 0, TAG 52:3; 0, TAG 54:4; 0, DAG 18:1; 0_18:2; 0, TAG 54:4; 0, DAG 18:1; 0_18:1; 0, and two highly correlated CE species CE 18:2; 0 and CE 16:0; 0, which are less correlated to the other five species (pairwise Pearson correlation *r* < 0.26), were significant. For AMD, five highly correlated PE species: PE 16:0; 0_20:4; 0, PE 18:0; 0_20:4; 0, PE 18:0; 0_18:2; 0, PE 16:0; 0_18:2; 0, and PE 18:1; 0_18:1; 0 (*r* > 0.5) reached significant results. When inspecting the forest plots showing the causal effect of each SNP, we observe that the SNPs with significant causal effects with the same effect direction as the MR result are all located in the *LIPC* locus. A previous study by Cadby et al.^33^ performed MR analysis of total PE on coronary artery disease, including only *LIPC* variants, and observed a slight risk-increasing effect. For comparison, we performed univariate MR analyses of PE species on IHD using only *LIPC* variants (Supplementary Data S2). Only PE 16:0; 0_20:4; 0 reached *P* < 0.05 in IVW-MR. Since we obtained significant MR results for PE species on AMD when including all instruments, we also performed MR analysis using only *LIPC* variants for PE species and AMD (Supplementary Data S2). We found a strong protective effect of PE 16:0; 0_20:4; 0 on AMD compared to the slight risk-increasing effect on IHD (Supplementary Fig. S4). The four other PE species also reached significance for AMD (Supplementary Fig. S5).

For cholelithiasis, PC 16:0; 0_20:4; 0, PC 17:0; 0_20:4; 0, PC 18:0; 0_20:4; 0, CE 20:4; 0, and PE 16:0; 0_20:4; 0 yielded significant results. All of these species reach the more stringent *P*-value threshold. The PC species and the CE species are highly correlated (*r* > 0.72), whereas the PE species is less correlated to the other species (*r* = 0.2).

### Multivariable MR

To understand if the lipid species reaching significance in univariable MR could be causal for the disease independent of the standard lipids we performed multivariable MR (MVMR) for each combination of one disease among IHD, AMD, and cholelithiasis, one standard lipid and one lipid species reaching *P* < 0.05/22 in univariable IVW MR, and *P* < 0.05 and consistent effect direction for all univariable MR methods for the disease (Supplementary Data S2). We focus on the standard lipid reported as an independent risk factor by previous studies, namely ApoB for IHD,^42^^,^^43^ HDL-C for AMD,^44^ and TC for cholelithiasis.^45^ The MVMR-IVW estimates are visualised in Fig. 6. The MVMR results for exploratory analyses including other standard lipids are shown in Supplementary Fig. S6.

For IHD, we observe a statistically significant effect for ApoB in MVMR independently of lipid species. A recent MR study compared the effects of non-HDL-C (TC minus HDL-C) and ApoB on coronary artery disease and found that non-HDL-C captures the contribution of ApoB-containing particles to disease risk even better than the ApoB particle concentration.^79^ In the MVMR analysis of lipid species and non-HDL-C as exposures and IHD as the outcome, we observed significant results for non-HDL-C but not for the lipid species. MVMR analyses including lipid species, ApoB, and non-HDL-C as exposures did not lead to significant results due to the high correlation (*r* = 0.96) between ApoB and non-HDL-C (Supplementary Data S2).

For AMD, we observed significant MVMR estimates for PEs independently of HDL-C. The PE species also remained significant when other standard lipids were included in the model.

For cholelithiasis, we observed significant MVMR estimates for CE 20:4; 0 and three PCs independently of TC. These lipids also reached significance in MVMR analyses when other standard lipids except LDL-C were included in the model. When LDL-C was included in the model neither LDL-C nor any lipid species reached significance.

We applied several robust MVMR methods to adjust for heterogeneity and pleiotropy. These methods provided similar effect sizes, and slightly larger standard errors compared to the multivariable IVW-MR (Supplementary Data S2).

We performed additional MVMR analyses for the three diseases and included as exposures one lipid species together with three standard lipids HDL-C, LDL-C, and TG. For IHD, we observed significant results for LDL-C but the large number of exposures led to weak instruments (conditional F-statistics <10) and therefore the results of this analysis might not be reliable. For AMD, conditional F-statistic were >10 and PEs were significant. For cholelithiasis, MV-IVW reported very high heterogeneity and no exposure reached significance.

## Discussion

In this study, we examined the link between 179 lipid species and 7 diseases using genetic predictors. First, we utilised genetic loci with a high impact on the plasma lipidome as predictors since at these loci the statistical power to detect disease association is the largest if the lipid species has a causal effect on the disease. We identified 11 high-impact loci based on lipid species GWAS in 7174 Finnish participants.^36^ We observed high pleiotropy among the high-impact loci, with 10 out of the 11 loci associated with >8 lipid species. This is in line with a meta-analysis of metabolites measured by nuclear magnetic resonance (NMR) in 136,000 individuals, which found that lipid, lipoprotein, and fatty acid trait-associated regions are more pleiotropic than those of the non-lipid traits since genes often affect several particle categories involved in lipoprotein metabolism.^35^ We found that for 79% of the trait-locus combinations, the lead variant alone explains a similar amount of heritability as the whole locus. The remaining 21% of trait-locus combinations have up to 8 independent genetic association signals, which makes these more informative for MR studies.

Second, we assessed whether PGS of lipid species derived from our study outperformed high-impact loci as genetic predictors. We found that PGS do not lead to notably better predictive performance compared to utilising independent signals of the high-impact loci. For PGS, variance explained (R^2^) was over 4% for 34 lipid species with the largest R^2^ reaching 16.9% for CE 20:4; 0. However, after excluding the high-impact loci from the PGS calculation, the variance explained was largely reduced with the largest R^2^ value dropping to 5.2%. The recent large meta-analysis of NMR metabolites^35^ in 136,000 individuals showed high polygenicity for lipid, lipoprotein, and fatty acid traits, with only 7.5% of metabolites being associated with <20 loci. The maximum number of associated loci per trait in our study was 7, and 30% (54/179) of lipid species were associated with 3–7 loci. While a strength of our study is a large sample size among the mass spectrometry-based metabolite GWAS, we are still limited in the sample size when it comes to the performance of PGS.

Another strength of our study is the Finnish genetic background of our study population that allows us to capture some variants that are enriched in the Finnish population but are extremely rare outside of Finland. The signals of the high-impact loci include three missense variants > two-fold enriched in the Finnish population: rs61738161 for *SPTLC3* associated with four Cers at locus *LINC01722*, rs113298164 for *LIPC* associated with PC O-16:2; 0/18:0; 0 and five PEs, and rs17159388 for *CERS4* associated with four SMs. On the other hand, a limitation of our study is that we do not have a non-Finnish replication cohort to assess the generalisability of our results to other genetic backgrounds.

Using disease information from FinnGen, UKBB, and Generation Scotland, we found that variants within the high-impact loci are associated with all seven diseases we studied: AD, AMD, cholelithiasis, IHD, MASLD, pure hypercholesterolaemia, and T2D.

We conducted MR analyses to assess whether lipid species impact disease risk. For MR instrument selection, we employed a genome-wide approach based on statistical significance among the LD-independent SNPs. Recently, a biologically driven strategy for MR instrument selection has been introduced.^80^ Another proposed approach is drug target MR, which utilises genetic variants within or near a gene encoding a druggable protein as instruments.^81^^,^^82^ A limitation of our study is that since we are unable to identify definite causal genetic variants or genes for individual lipid species, we cannot use the biologically driven instrument selection. Another challenge in applying drug target MR is the limited statistical power to identify robust genetic instruments near the known drug targets.

The *APOE* locus is well-known to be highly pleiotropic.^83^ The locus encodes a set of apolipoproteins involved in lipoprotein particle trafficking in various tissues and cells and plays a key role as a lipid transport vesicle in cerebral spinal fluid.^84^ We observed associations between the *APOE* locus and all seven diseases. It is the only locus reaching the Bonferroni-corrected significance threshold for T2D and AD. For AD, no lipid species remained significant in our univariable MR analyses, and for T2D, only PE 16:0; 0_20:4; 0 and SM 36:1; 2 reached significance, but *P*-values were close to the significance thresholds. For MASLD, no locus reached the Bonferroni-corrected significance threshold, and no lipid species was significant in the MR analysis.

Across the high-impact loci, we observed nine independent genetic associations for pure hypercholesterolaemia. Because of the obvious direct relationship with cholesterol levels, we focus here on the MR results of the remaining diseases, IHD, AMD, and cholelithiasis.

In addition to *APOE*, the high-impact loci *CERS4*, and *ZPR1* are linked to IHD. *CERS4* encodes a ceramide synthase which produces ceramides from sphingosine and a long chain acyl CoA^85^ and had high heritability for SMs in our study. The candidate genes at the *ZPR1* locus are among the *APOA5/APOA4/APOA1/APOC3* gene cluster which encodes apolipoproteins involved in apolipoprotein particle trafficking.^34^ We observed high heritability for TAG and DAG species at the *ZPR1* locus. Univariable MR analyses on IHD showed potential causal effects for TAG, DAG, and CE species but MVMR analyses implicated that ApoB, or non-HDL-C that is highly correlated (*r*^2^ = 0.96) with ApoB, is a better candidate to be a causal risk factor than the lipid species. Thus, our MR analyses support the causal role of elevated ApoB/non-HDL-C on IHD risk reported previously by MVMR studies of standard lipids^42^^,^^43^ and by a MVMR study including NMR metabolites.^86^ These results are in line with the European Society of Cardiology/European Atherosclerosis Society 2019 guidelines for the management of dyslipidemias to prevent cardiovascular disease, which recommend measuring ApoB in addition to LDL-C.^87^ However, we acknowledge that a MVMR analysis can only compare the potential causality between the traits that are available in the study. There always remains a possibility that some unmeasured molecular quantity could be an even better candidate for having a causal effect on the outcome than any of the available measurements. Additionally, we have little statistical power to tease apart the effects of highly correlated pairs of exposures, such as ApoB and non-HDL-C.

For the *LIPC* region, three independent association signals have been reported for the standard lipids^19^ and an additional signal has been discovered for lipid species.^33^^,^^36^ At this locus, we observed large heritability for PEs (6–16%), with the largest heritability found for PE 16:0; 0_20:4; 0. The strong association with PEs was also discovered by Cadby et al.,^33^ who noted that these lipid species were direct substrates for hepatic lipase and reported a slight risk-increasing effect of total PE on coronary artery disease in MR analysis including only *LIPC* variants (Beta = 0.06, *P* = 6e-9). In a univariate MR analysis for IHD using only *LIPC* variants, we observed consistent effect sizes at nominal significance level for PE 16:0; 0_20:4; 0 (Beta = 0.03, *P* = 0.02). Interestingly, for AMD, we found a strong protective effect of PE 16:0; 0_20:4; 0 (Beta = −0.27, *P* = 6e-32) when utilising only *LIPC* variants, and a similar effect on AMD was observed for the other four PEs measured.

Even though the protective effect of five PEs on AMD remained significant when all genetic instruments were considered, the causal effect estimates of the genetic instruments show that the result is strongly driven by the *LIPC* variants (Supplementary Fig. S2). This suggests that the AMD risk is rather impacted by an *LIPC*-dependent pathway than by the PEs in general.

The apparent protective effect of PEs on AMD was also observed by Yin et al.,^48^ who hypothesised that the putative causal effect of *LIPC* expression on AMD is exerted through the glycerophospholipid metabolic pathway. Another hypothesis given in a recent review is that *LIPC* variants might exert specific effects on lipid metabolism locally in the retina.^38^

A previous MR analysis of standard lipids has suggested HDL-C as a causal risk factor for AMD^44^ and also reported heterogeneity in AMD risk between lipid-increasing alleles in *LIPC* compared to elsewhere in the genome. In our univariable MR, HDL-C did not reach significance (*P* = 0.066), which might be due to the lower sample size compared to the previous MR analysis, which utilised lipid GWAS of over 189,000 individuals.^44^ In our MVMR, the effects of the PEs on AMD remained significant after the inclusion of HDL-C in the analysis.

The high-impact locus *ABCG8* is specifically strongly associated with CE 20:2; 0 (regional heritability = 4%), as reported by two previous studies.^20^^,^^33^ The lead variant rs4245791-T is associated with lower CE levels and a higher risk of cholelithiasis (gallstones) in FinnGen. Variants in *ABCG8*, such as the missense variant rs11887534, have been linked to cholelithiasis by several studies.^88^, ^89^, ^90^ It has been hypothesised that rs11887534-C increases the efficiency of the cholesterol transport into the bile lumen, which causes cholesterol hypersaturation of the bile and thereby increases the risk of cholesterol gallstones.^88^ In line with this interpretation, the observed association between rs11887534-C with lower CE levels could be due to lowered cholesterol levels in plasma caused by increased transport into bile.

The univariable MR analyses on cholelithiasis provided significant results for five species of the classes PC, CE, and PE. Interestingly, all those species contain arachidonic acid (20:4). This is in line with univariable MR results from the Finnish METSIM cohort where two PCs containing arachidonic acid^48^ and campesterol^47^^,^^48^ reached significance for gallbladder disorders (Supplementary Data S3).

It remains uncertain if standard lipids are an independent risk factor for cholelithiasis. In a recent study, TC was significant in MVMR independently of LDL-C^45^ but a study for the related trait cholecystitis reported a significant effect of LDL-C and not TC.^46^ In our analyses, TC was significant in univariable MR, and the effects of the three PCs, the CE but not the PE species remained significant after the inclusion of TC in MVMR analyses.

Our study shows how we can utilise genetic predictors of lipid species to gain insights into disease risk. We have observed that ApoB remains the candidate for having a causal effect on IHD even when we account for more detailed lipid species in multivariate analysis. Additionally, we have verified a clear heterogeneity in the AMD risk among genetic variants that affect PE species, pointing out the special pattern observed in the *LIPC* gene. For cholelithiasis, MVMR suggested a protective effect of four lipid species containing arachidonic acid independently of TC. While our study is large among the existing genetic studies of the lipidome, larger sample sizes are needed to achieve stronger genetic predictors of lipid species and more independent genetic instruments to be used in the MR framework.

## Contributors

L.O., R.T., M.J.G., K.S., S.R., V.V., C.H., and M.P. conceived and designed the study; L.O. performed Generation Scotland GWAS, Mendelian randomisation analyses and all statistical analyses and reported the results; R.T. performed GeneRISK lipid species GWAS; D.L.M. extracted individual IDs for disease cases for Generation Scotland GWAS, S.E.R. performed quality control of GeneRISK genotype data; L.O., R.T., M.J.G. E.W., K.S., S.R., V.V., C.H., and M.P. interpreted the results; M.J.G., C.K. and K.S. performed lipidomic profiling and processed the raw data; L.O. drafted the manuscript with help from R.T., V.V., C.H., and M.P; R.T., V.V., C.H., and M.P. supervised the study. L.O. and R.T. accessed and verified the data. L.O., R.T., and M.P. were responsible for the decision to submit the manuscript. FinnGen contributed disease GWAS utilised in the PheWAS and Mendelian randomisation analyses. All authors read, commented, and approved the manuscript.^8^

## Data sharing statement

Individual-level data are not publicly available due to legal privacy limitations. Access can be obtained through individual participating biobanks. DNA, blood, serum, and plasma samples of GeneRISK study participants, in addition to their demographic information and health data, are stored in the THL Biobank (https://thl.fi/en/research-and-development/thl-biobank/for-researchers/sample-collections/generisk-study). FINRISK samples are stored in the THL Biobank (https://thl.fi/en/research-and-development/research-and-projects/the-national-finrisk-study). GeneRISK, FINRISK, and FinnGen data are available under restricted access via procedures outlined in the Finnish Biobank Act and access can be obtained through Finnish biobanks’ FinBB portal (www.finbb.fi; email: info@fingenious.fi) UK Biobank data are available through a procedure described at https://www.ukbiobank.ac.uk/enable-your-research. Generation Scotland data may be accessed through an application process described at https://genscot.ed.ac.uk/for-researchers/access.

Summary level data are reported in Supplementary Data.

GeneRISK lipid species GWAS summary statistics are available on GWAS catalogue (https://www.ebi.ac.uk/gwas/) under accession codes GCST90277238-GCST90277416.

GWAS summary statistics for UK Biobank (UKBB) were obtained from the Pan-UKBB project (https://pan.ukbb.broadinstitute.org/). FinnGen disease GWAS from release 12 were utilised in this study, summary statistics are available from https://www.finngen.fi/en/access_results.

## Declaration of interests

K.S. is CEO of Lipotype GmbH. K.S. and C.K. are shareholders of Lipotype GmbH. M.J.G. is an employee of Lipotype GmbH. D.L.M. is an employee of Optima Partners Ltd. The FinnGen project is funded by two grants from Business Finland (HUS 4685/31/2016 and UH 4386/31/2016) and the following industry partners: AbbVie Inc., AstraZeneca UK Ltd, Biogen MA Inc., Bristol Myers Squibb (and Celgene Corporation & Celgene International II Sàrl), Genentech Inc., Merck Sharp & Dohme LCC, Pfizer Inc., GlaxoSmithKline Intellectual Property Development Ltd., Sanofi US Services Inc., Maze Therapeutics Inc., Janssen Biotech Inc, Novartis AG, and Boehringer Ingelheim International GmbH. The remaining authors declare no competing interests.

## Appendix ASupplementary data

Supplementary Data 3

Supplementary Note 1
