# Supplementary material for: Examining the link between 179 lipid species and 7 diseases using genetic predictors
Source: eBioMedicine. 2025 Mar 28;114:105671. doi: 10.1016/j.ebiom.2025.105671 (PMC11995710; doi:10.1016/j.ebiom.2025.105671)
Supplement: Supplementary Note 1 [file mmc4.docx]

**Supplementary Note 1**

**Supplementary Methods**

**GeneRISK**

Participants were recruited from the Kymenlaakso province in South-Eastern Finland by identifying 4,857 individuals from the population register at random and inviting them by mail. Further, 1,369 individuals were recruited from customers of Helsinki and Turku offices of a private and occupational health care provider. Additionally, online advertising was used to recruit 1,116 blood donors. Individuals under guardianship, with a previous history of Atherosclerotic Cardiovascular Disease, and pregnant women were excluded from the study.

**FinnGen**

FinnGen^1^ is a random sample of subjects from Finnish clinical and population-based biobanks. The ethics statement and Supplemental Acknowledgements for FinnGen are listed at the end of this Supplementary Note. Funding information for FinnGen is listed in the Declaration of Interests section of the manuscript. FinnGen consists of legacy cohorts with genotypes already available and prospective samples. Genotyping of prospective samples was performed with the ThermoFisher Axiom custom array which tags 655,973 variants. The Array Power Tools software was used for genotype calling. Legacy cohorts were genotyped using various Illumina arrays. Genotype calling was performed using GenCall or zCall algorithms. Samples were removed if

- Pihat was > 0.9 and samples were not monozygotic or replicates
- A discrepancy between genetically determined sex and reported sex was found (F-value ≤ 0.3 for females and ≥ 0.8 for males)
- Missingness was ≥ 5%
- Heterozygosity was ±4 standard deviations from the population average
- Pihat was > 0.1 with ≥ 14 samples
- Samples were ±4 standard deviations away from the population average based on the first two genetic principal components.

Samples with evidence of a Mendelian error or containing replicate samples with > 50,000 discrepancies were tagged. Variants with a call rate < 98% and those failing the Hardy-Weinberg Equilibrium test (*P*-value < 1e-6) were removed.

Genetic ancestry was estimated by combining samples with the 1000 Genomes Project dataset^2^. Genetic principal components were calculated using 180,042 pruned SNPs. Samples deviating from the main cluster (*N* = 15,898) were removed. Another PCA was computed from the remaining samples. North-Western European and Finnish 1000 Genome Project samples were projected onto the space generated by the first 3 principal components. The centroid of each cluster was calculated and used to calculate the squared Mahalanobis distances for each FinnGen sample against each of the centroids. The squared distance was mapped into a probability and samples were retained if there was ≥ 95% probability of belonging to the Finnish ancestry cluster. This method identified further 879 outliers.

The FinnGen release 12 consisting of a total of 500,348 samples (282,064 female sex, 218,284 male sex) were used for GWAS analysis.

GWAS were performed with regenie v2.2.4^3^. For regenie step 1 LOCO prediction computation, age, sex, the first 10 principal components, FinnGen 1 or 2 chip, and legacy genotyping batch were used as covariates. Covariates with < 10 cases were excluded. For genetic relatedness calculation in regenie step 1, variants with MAF > 1%, imputation INFO score > 0.95 in all batches, and > 97% non-missing genotypes were included. The variants were LD pruned with an r^2^ threshold of 0.2 and window size of 1.5 Mb. A set of 188,153 variants remained for relatedness calculation. A genotype block size of 1,000 was used in regenie step 1. Association tests were run for each endpoint for each variant with a minimum allele count of 5 among each endpoint’s cases and controls. For variants with an initial *P*-value < 0.01 the approximate Firth test was used to compute the standard error based on effect size and likelihood ratio test *P*-value. Genomic positions are on GRCh38.

**UK Biobank**

UK Biobank (UKBB) is a population-based study. Publicly available summary statistics from the Pan-UKBB project^4^ (<https://pan.ukbb.broadinstitute.org/>). were utilised in this study. Ancestry was genetically estimated by performing PCA on individuals from the 1000 Genomes Project + Human Genome Diversity Panel combined reference dataset and then projecting UK Biobank individuals into the same principal component space. More details are provided on [https://pan.ukbb.broadinstitute.org/docs/qc#ancestry-definitions](https://pan.ukbb.broadinstitute.org/docs/qc#ancestry-definitions). GWAS were run separately for each ancestry. In this study, we utilised GWAS of individuals of European ancestry.

GWAS analyses were conducted with the mixed-model logistic regression method SAIGE^5^ adjusting for age, sex, age*sex, age^2^, age^2^*sex, and the first 10 principal components. Participants with European ancestry (*N* = 420,531), and of the 97,059,328 imputed variants from the UKBB version 3, 29,865,259 variants with INFO scores > 0.8 were included. Additionally, variants with an allele count < 20 in the European population were excluded. We lifted genomic positions over from GRCh37 to GRCh38 using liftOver^6^ and retained variants included in the FinnGen GWAS.

**Generation Scotland**

Generation Scotland: Scottish Family Health Study (GS:SFHS)^7^, is a family-based study of about 24,000 volunteers across Scotland aged between 18-99 years. Ethical approval for the GS:SFHS study was obtained from the Tayside Committee on Medical Research Ethics (on behalf of the National Health Service). Phenotypic data and whole blood samples for DNA quantification were obtained at baseline. Genotype data was assayed for 20,195 individuals in two batches with 9863 individuals in the first batch and the remainder in the second batch. Genotyping was performed using the Illumina HumanOmniExpressExome-8 v1.0 BeadChip and the Illumina HumanOmniExpressExome-8 v1.2 BeadChip, respectively. SNPs with Hardy-Weinberg *P*-value < 1e-6 were removed. SNPs or individuals with a call rate < 98% were removed. Individual-level genotypes at erroneous SNPS were set to missing to remove Mendelian errors. Individuals with a discrepancy between genetically determined sex and reported sex were removed from the cohort.

Imputation was performed with the HRC panel v1.1. Consistency with the reference panel was ensured by checking autosomal haplotypes for strand orientation, reference allele, and position. Pre-phasing was performed using Shapeit2 v2r837^8,9^. The Shapeit2 duohmm option11^10^ and cohort family structure were used to improve imputation quality^11^. Monogenic variants and variants with low imputation quality (INFO < 0.4) were removed. For GWAS analysis 24,111,857 variants remained.

Ancestry outliers were removed from the dataset. These were defined as individuals more than six standard deviations away from the mean in a PCA of Generation Scotland merged with 1092 participants from the 1000 Genomes Project.

GWAS were performed with the mixed-model logistic regression method SAIGE using age, sex, and the first 10 principal components as covariates. The genomic positions were lifted from GRCh37 to GRCh38 using liftOver^6^ and the variants included in the FinnGen GWAS were retained.

**Supplemental references**

1. Kurki, M. I. *et al.* FinnGen provides genetic insights from a well-phenotyped isolated population. *Nature* **613**, 508–518 (2023).

2. Auton, A. *et al.* A global reference for human genetic variation. *Nature* **526**, 68–74 (2015).

3. Mbatchou, J. *et al.* Computationally efficient whole-genome regression for quantitative and binary traits. *Nat Genet* **53**, 1097–1103 (2021).

4. Karczewski, K. J. *et al.* Pan-UK Biobank GWAS improves discovery, analysis of genetic architecture, and resolution into ancestry-enriched effects. 2024.03.13.24303864 Preprint at https://doi.org/10.1101/2024.03.13.24303864 (2024).

5. Zhou, W. *et al.* Efficiently controlling for case-control imbalance and sample relatedness in large-scale genetic association studies. *Nat Genet* **50**, 1335–1341 (2018).

6. Kuhn, R. M., Haussler, D. & Kent, W. J. The UCSC genome browser and associated tools. *Brief Bioinform* **14**, 144–161 (2013).

7. Smith, B. H. *et al.* Cohort Profile: Generation Scotland: Scottish Family Health Study (GS:SFHS). The study, its participants and their potential for genetic research on health and illness. *Int J Epidemiol* **42**, 689–700 (2013).

8. Delaneau, O., Marchini, J. & Zagury, J.-F. A linear complexity phasing method for thousands of genomes. *Nat Methods* **9**, 179–181 (2012).

9. Delaneau, O., Zagury, J.-F. & Marchini, J. Improved whole-chromosome phasing for disease and population genetic studies. *Nat Methods* **10**, 5–6 (2013).

10. O’Connell, J. *et al.* A General Approach for Haplotype Phasing across the Full Spectrum of Relatedness. *PLoS Genet* **10**, e1004234 (2014).

11. Durbin, R. Efficient haplotype matching and storage using the positional Burrows-Wheeler transform (PBWT). *Bioinformatics* **30**, 1266–1272 (2014).

**FinnGen ethics statement**

Patients and control subjects in FinnGen provided informed consent for biobank research, based on the Finnish Biobank Act. Alternatively, separate research cohorts, collected prior the Finnish Biobank Act came into effect (in September 2013) and start of FinnGen (August 2017), were collected based on study-specific consents and later transferred to the Finnish biobanks after approval by Fimea (Finnish Medicines Agency), the National Supervisory Authority for Welfare and Health. Recruitment protocols followed the biobank protocols approved by Fimea. The Coordinating Ethics Committee of the Hospital District of Helsinki and Uusimaa (HUS) statement number for the FinnGen study is Nr HUS/990/2017.

The FinnGen study is approved by Finnish Institute for Health and Welfare (permit numbers: THL/2031/6.02.00/2017, THL/1101/5.05.00/2017, THL/341/6.02.00/2018, THL/2222/6.02.00/2018, THL/283/6.02.00/2019, THL/1721/5.05.00/2019 and THL/1524/5.05.00/2020), Digital and population data service agency (permit numbers: VRK43431/2017-3, VRK/6909/2018-3, VRK/4415/2019-3), the Social Insurance Institution (permit numbers: KELA 58/522/2017, KELA 131/522/2018, KELA 70/522/2019, KELA 98/522/2019, KELA 134/522/2019, KELA 138/522/2019, KELA 2/522/2020, KELA 16/522/2020), Findata permit numbers THL/2364/14.02/2020, THL/4055/14.06.00/2020,,THL/3433/14.06.00/2020, THL/4432/14.06/2020, THL/5189/14.06/2020, THL/5894/14.06.00/2020, THL/6619/14.06.00/2020, THL/209/14.06.00/2021, THL/688/14.06.00/2021, THL/1284/14.06.00/2021, THL/1965/14.06.00/2021, THL/5546/14.02.00/2020, THL/2658/14.06.00/2021, THL/4235/14.06.00/202, Statistics Finland (permit numbers: TK-53-1041-17 and TK/143/07.03.00/2020 (earlier TK-53-90-20) TK/1735/07.03.00/2021, TK/3112/07.03.00/2021) and Finnish Registry for Kidney Diseases permission/extract from the meeting minutes on 4^th^ July 2019.

The Biobank Access Decisions for FinnGen samples and data utilized in FinnGen Data Freeze 9 include: THL Biobank BB2017_55, BB2017_111, BB2018_19, BB_2018_34, BB_2018_67, BB2018_71, BB2019_7, BB2019_8, BB2019_26, BB2020_1, Finnish Red Cross Blood Service Biobank 7.12.2017, Helsinki Biobank HUS/359/2017, HUS/248/2020, Auria Biobank AB17-5154 and amendment #1 (August 17 2020), AB20-5926 and amendment #1 (April 23 2020) and it´s modification (Sep 22 2021), Biobank Borealis of Northern Finland_2017_1013, Biobank of Eastern Finland 1186/2018 and amendment 22 § /2020, Finnish Clinical Biobank Tampere MH0004 and amendments (21.02.2020 & 06.10.2020), Central Finland Biobank 1-2017, and Terveystalo Biobank STB 2018001 and amendment 25^th^ Aug 2020.

**Supplemental acknowledgments**

We want to acknowledge the participants and investigators of FinnGen study. Following biobanks are acknowledged for delivering biobank samples to FinnGen: Auria Biobank (www.auria.fi/biopankki), THL Biobank (www.thl.fi/biobank), Helsinki Biobank (www.helsinginbiopankki.fi), Biobank Borealis of Northern Finland (<https://www.ppshp.fi/Tutkimus-ja-opetus/Biopankki/Pages/Biobank-Borealis-briefly-in-English.aspx>), Finnish Clinical Biobank Tampere (www.tays.fi/en-US/Research_and_development/Finnish_Clinical_Biobank_Tampere), Biobank of Eastern Finland (www.ita-suomenbiopankki.fi/en), Central Finland Biobank (www.ksshp.fi/fi-FI/Potilaalle/Biopankki), Finnish Red Cross Blood Service Biobank ([www.veripalvelu.fi/verenluovutus/biopankkitoiminta](http://www.veripalvelu.fi/verenluovutus/biopankkitoiminta)), Terveystalo Biobank ([www.terveystalo.com/fi/Yritystietoa/Terveystalo-Biopankki/Biopankki/](http://www.terveystalo.com/fi/Yritystietoa/Terveystalo-Biopankki/Biopankki/)) and Arctic Biobank (<https://www.oulu.fi/en/university/faculties-and-units/faculty-medicine/northern-finland-birth-cohorts-and-arctic-biobank>). All Finnish Biobanks are members of BBMRI.fi infrastructure ([www.bbmri.fi](http://www.bbmri.fi/)). Finnish Biobank Cooperative -FINBB (<https://finbb.fi/>) is the coordinator of BBMRI-ERIC operations in Finland. The Finnish biobank data can be accessed through the Fingenious^®^ services (<https://site.fingenious.fi/en/>) managed by FINBB.
