# Supplementary material for: Examining the link between 179 lipid species and 7 diseases using genetic predictors
Source: eBioMedicine. 2025 Mar 28;114:105671. doi: 10.1016/j.ebiom.2025.105671 (PMC11995710; doi:10.1016/j.ebiom.2025.105671)
Supplement: Supplementary Figs. S1–S6 [file mmc6.docx]

**
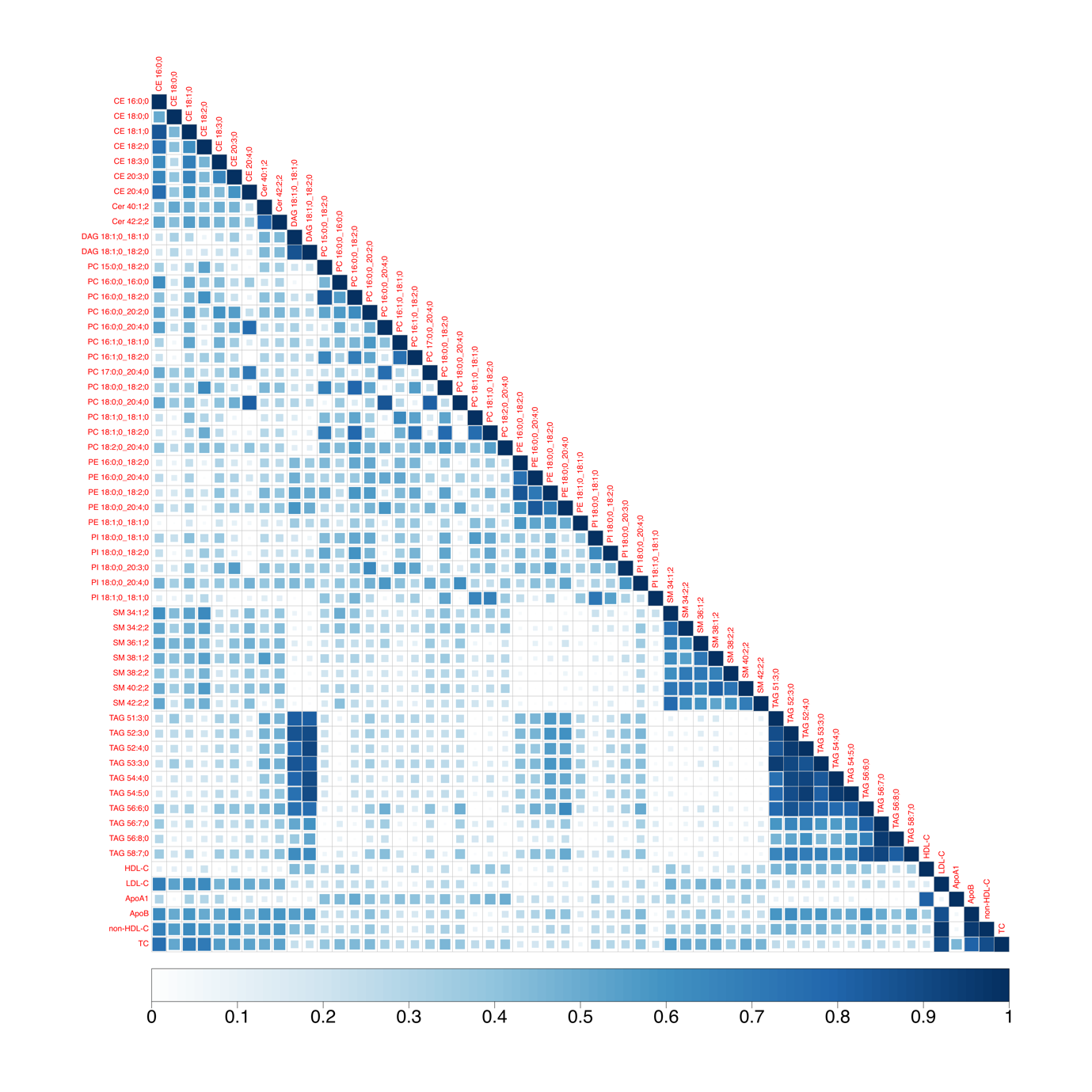
**

**Supplementary Figure 1. Heatmap of the absolute pairwise Pearson correlation between the 57 lipid species or standard lipids measured in GeneRISK and included in MR analyses.**

**Supplementary Figure 2. Significant MR results for IHD, AMD, and Cholelithiasis.** Left panel: Scatterplot of SNP effects on the exposure (lipid species) against SNP effects on the outcome (disease). Right panel: Forest plot comparing the causal effect of using each of the SNPs on their own (Wald ratio) to two methods combining all SNPs. *P*-values for MR tests are given for different methods in the legend of the scatter plot, in addition to the intercept term of MR Egger analysis. 95% confidence intervals are shown. Plots were created with ggplot2 R package using code adapted from the **TwoSampleMR R package.**

**Supplementary Figure 3. Regional heritability (h^2^) of standard lipids for genome-wide significant loci estimated by FINEMAP.** High-impact loci (h^2^>2% for at least one lipid species) are coloured, and the other genome-wide significant loci not among the high-impact loci are depicted as grey.

**Supplementary Figure 4. MR results of PE 16:0;0_20:4;0** **for IHD and AMD utilizing only *LIPC* variants.** Left panel: Scatterplot of SNP effects on the exposure (lipid species) against SNP effects on the outcome (disease). Right panel: Forest plot comparing the causal effect of using each of the SNPs on their own (Wald ratio) to two methods combining all SNPs. *P*-values for MR tests are given for different methods in the legend of the scatter plot, in addition to the intercept term of MR Egger analysis. 95% confidence intervals are shown. Plots were created with ggplot2 R package using code adapted from the **TwoSampleMR R package.**

**Supplementary Figure 5. MR results of PE species (excluding PE 16:0;0_20:4;0)** **and AMD utilizing only *LIPC* variants.** Left panel: Scatterplot of SNP effects on the exposure (lipid species) against SNP effects on the outcome (disease). Right panel: Forest plot comparing the causal effect of using each of the SNPs on their own (Wald ratio) to two methods combining all SNPs. *P*-values for MR tests are given for different methods in the legend of the scatter plot, in addition to the intercept term of MR Egger analysis. 95% confidence intervals are shown. Plots were created with ggplot2 R package using code adapted from the **TwoSampleMR R package.**

**Supplementary Figure 6. Forest plots of effect sizes of IVW-MVMR for IHD, AMD, and Cholelithiasis.** Each multivariable MR (MVMR) analysis contains as exposures a standard lipid (Exposure 1, in red) and a lipid species (Exposure 2, in black). MVMR results are shown for the lipid species reaching significance in univariable MR. The pairwise Pearson correlation of the exposure with the standard lipid included is listed. Univariable IVW-MR *P*-value for the standard lipid included in the model are listed in brackets behind the standard lipid name. For TG, univariable MR is not part of the main MR results, due to a low number of instruments (3), but was performed as an additional analysis (Supplementary Data 2). MVMR results for models including the standard lipid reported to be causal for the disease are shown in Figure 6.
